# Percutaneous Mitral Valve Repair: Multi-Modality Cardiac Imaging for Patient Selection and Intra-Procedural Guidance

**DOI:** 10.3389/fcvm.2019.00142

**Published:** 2019-09-20

**Authors:** Omar K. Khalique, Rebecca T. Hahn

**Affiliations:** Division of Cardiology, Structural Heart and Valve Center, Columbia University Medical Center and New York Presbyterian Hospital, New York, NY, United States

**Keywords:** mitral regurgitation, percutaneous mitral annuloplasty, percutaneous edge-to-edge leaflet repair, multimodality cardiac imaging, interventional echocardiography

## Abstract

Percutaneous mitral valve repair is an important procedure for patients at high risk of surgical mitral valve repair. Multi-modality Cardiac Imaging plays a key role in these procedures. MitraClip is the first and most utilized percutaneous mitral repair device and experience is has grown to treat not only typical but atypical and complex lesions. Cardioband is an emerging percutaneous annuloplasty system with promising early results. This review will focus on the comprehensive multi-modality cardiac imaging for patient selection and intra-procedural guidance of the MitraClip and Cardioband systems.

## Introduction

Mitral regurgitation (MR) is one of the most common valvular diseases in the world. Percutaneous technologies have been increasingly investigated as an alternative to open heart surgery in high-surgical risk patients. Several mitral repair systems have been approved in the United States or Europe. The Mitraclip (Abbott Structural, Santa Clara, California) percutaneous edge-to-edge repair device has received Conformité Européenne (CE) mark approval for degenerative and functional MR in Europe as well as Food and Drug Administration (FDA) approval for degenerative and more recently functional MR in the United States. Cardioband (Edwards Lifesciences, Irvine, California) percutaneous annuloplasty system has received CE mark approval for functional MR in Europe. Cardiac imaging pre-procedural assessment and intra-procedural guidance are crucial for procedural success and will be reviewed here.

## Patient Selection

### Identification of Valve Morphology

Multiple Societal guidelines (1, 2) recommend identification of the etiology and consequence of MR as the initial step in evaluation. A recent American College of Cardiology (ACC) consensus statement (3) recommend the use of transthoracic echocardiography (TTE) for the initial evaluation of patients with signs or symptoms of MR. Identification of the etiology of MR (primary or secondary) as well as the hemodynamic effects of MR (i.e., on ventricular or atrial size and function) are essential for the selection of the appropriate patient for transcatheter repair procedures. Isolated annular devices are not appropriate for primary disease. Current Societal and FDA recommendations for use of the edge-to-edge repair system in degenerative disease (Class IIB) include patients who are at high surgical risk with Stage D qualifications including an effective regurgitant orifice area (EROA) of ≥40 mm^2^, regurgitant volume of ≥60 cc and regurgitant fraction of ≥50%. Importantly for chronic MR, the left ventricle (LV) should be dilated.

The recent ACC/American Heart Association guideline update (4) as well as the recent ASE updated guideline (5), recommend using the same quantitative criteria for primary as secondary MR to define severe disease: EROA ≥40 mm^2^, regurgitant volume of ≥60 cc. Although current European Society of Cardiology guideline (which uses a lower cut-off) (2) are not aligned with the European Society of Echocardiography guideline (which uses the aforementioned cut-offs) (6) both American and European guidelines recognized that worse outcomes for functional MR may be seen with an EROA of >20 mm^2^ (2, 4). FDA approval for use of the edge-to-edge repair device for functional MR was based on the results of the Cardiovascular Outcomes Assessment of the Mitraclip Percutaneous Therapy for Heart Failure Patients With Functional Mitral Regurgitation (COAPT) trial (7). The approved indication thus includes secondary or functional MR include high surgical risk patients with LV dysfunction, moderate-severe or severe MR (EROA ≥30 mm^2^), with LV dilatation (LV end-systolic dimension ≤ 70 mm) and LV ejection fraction >20%. Guidelines are expected to change based on the results of the randomized trial and these new indications.

## Mitral Valve Regurgitation Quantification

**Figure 1:**
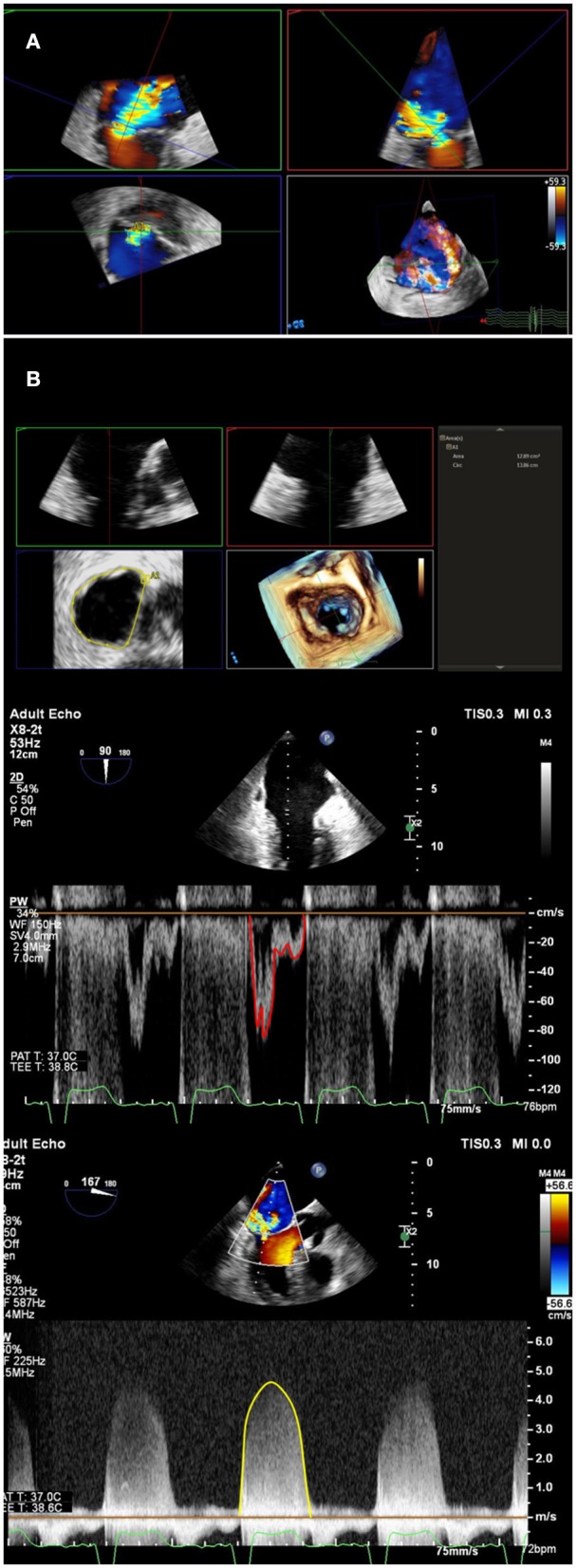
Multi-parametric assessment of mitral regurgitation. In addition to qualitative color Doppler grading, multiple methods of mitral regurgitant quantification should be used. **(A)** A multiplanar reconstruction of 3-Dimensional echocardiographic color Doppler can be used to directly planimeter vena contracta area. A double-oblique method is used to align long-axis images (upper left and right), and directly planimeter vena contracta area (lower left). **(B)** Mitral valve inflow stroke volume, which includes mitral regurgitant volume, can be calculated by multiplying mitral annulus area (top panel) by pulsed-wave velocity time integral at the annulus level (middle panel). Left ventricular or right ventricular outflow tract calculated stroke volume, in the absence of aortic or pulmonic insufficiency, can be subtracted from mitral stroke volume to obtain regurgitant volume. Regurgitant volume can be divided by mitral regurgitation continuous-wave Doppler velocity time integral (bottom panel) to calculate quantitative Doppler derived regurgitant orifice area.

Baseline MR should be quantified according to the guidelines described by the American Society of Echocardiography and European Association of Echocardiography (5, 6). A multi-parametric, multi-modality method should always be performed, using both qualitative and quantitative assessment of MR which are well-described in the guidelines. Color Doppler has been an easy and rapid parameter to assess MR severity, making use of the three components of regurgitant jet: proximal flow convergence dependent on both orifice and flow, the vena contracta which can approximate the regurgitation orifice, and jet area which may relate to regurgitant volume. However, the color Doppler parameters are dependent on technical and ultrasound physics parameters, the shape of the orifice as well as hemodynamic variables. Thus, color Doppler measurements are highly variable and may be used to detect the presence of MR but are not recommended as the sole method to document severity (6); the three components should be integrated to improve accuracy (5). As per the society guidelines, MR severity is based on quantitative parameters of regurgitant volume, regurgitant fraction, and EROA. These measurements can be performed by proximal Isovelocity surface area (PISA) method, quantitative Doppler and 3D color Doppler vena contracta planimetry (Figure 1). The pitfalls of each method are covered by the guidelines and are beyond the scope of this document (5, 6).

## Mitraclip

Percutaneous edge-to-edge repair with mitraclip is the most common percutaneous mitral repair technique performed worldwide. A recent multi-center clinical study of the German national patient sample included 13,575 implants over 5 years (8). Repair using this device mimics the Alfieri surgical repair. Patient selection and procedural guidance is largely predicated upon 2-dimensional (2D) and 3-dimensional (3D) echocardiographic imaging, particularly TEE. There is currently no role for multi-detector row computed tomography (MDCT) for pre-procedural screening for mitraclip (9).

### Procedural Planning

The Mitral Valve Academic Research Consortium (MVARC) definitions of “device success” measured at 30 days are listed in Table 1 (10, 11). The goal of the procedure is thus to reduce the mitral regurgitation to no greater than mild, recognizing that MR reduction is considered optimal when post-procedure MR is reduced to trace or absent. MR reduction is considered acceptable when post-procedure MR is reduced by at least 1 class or grade from baseline and to no more than moderate (2+) in severity. Recent outcomes data from the Society of Thoracic Surgeons/American College of Cardiology Transcatheter Valve Therapy Registry, showed that reduction to ≤ mild (grade 1) disease was dependent on experience (achieved in 66.5% of patients at sites with pre-commercial experience, compared to 57.4% at commercial sites, *p* = 0.04) (12). Sorajja et al. also showed that increased mortality was seen with >mild MR (13). The original EVEREST trial echocardiographic anatomic inclusion criteria included non-rheumatic valve morphology, mitral valve area ≥ 4 cm^2^, flail gap ≤ 10 mm, flail width ≤ 15 mm, coaptation depth ≤ 11 mm, coaptation length ≥ 2 mm, and central regurgitation at the A2-P2 interface (29). Case reports, observational studies, and clinical experience have since shown the possibility of successful therapy outside of these original criteria (14–16). Lubos et al. found that effective regurgitant orifice area >70.8 mm^2^ and mitral valve area ≤ 3.0 cm^2^ independently predicted clip failure (defined by aborted procedure or inability to reduce MR to ≤ 2+ in severity) (33). A recent paper studying treatment of degenerative MR suggested that higher baseline left-ventricular end-diastolic diameter and mitral annular diameter predict greater than mild residual MR after mitraclip (17). In current clinical practice, absolute anatomic limitations are very few (18). Hahn et al. listed the echocardiographic features associated with ideal, challenging and difficult anatomies (Table 2) (18). Severe calcification of nearly the entire leaflet length at the grasping zone, short leaflet length and low baseline mitral valve area (MVA) are the most common current contraindications. Prior research has shown that mitral valve area decreases by ~50% after a single Mitraclip is placed (19–21). Thus, in general, a baseline MVA >4.0 cm^2^ is desirable. However, a smaller baseline area may be acceptable in patients with small body habitus if potential benefits outweigh risks.

**Table 1:** Definition of device success: This table lists the definition of device success as outlined by Mitral Valve Academic Research Consortium Stone et al. (10).

Device success (measured at 30 days and at all later post-procedural intervals)
All of the following must be present:
I. Absence of procedural mortality or stroke; and
II. Proper placement and positioning of the device; and
III. Freedom from unplanned surgical or interventional procedures related to the device or access procedure; and
IV. Continued intended safety and performance of the device, including:
a. No evidence of structural or functional failure
b. No specific device-related technical failure issues and complications
c. Reduction of mitral regurgitation to either optimal or acceptable levels^*^
without significant mitral stenosis (i.e., post-procedure mitral valve area is ≥1.5 cm^2^ with a transmitral gradient <5 mm Hg), and with no greater than mild (1+) para-device mitral regurgitation and without associated hemolysis)

* *Mitral regurgitation reduction is considered optimal when post-procedure mitral regurgitation is reduced to trace or absent. mitral regurgitation reduction is considered acceptable when post-procedure mitral regurgitation is reduced by at least 1 class or grade from baseline and to no more than moderate (2+) in severity*.

**Table 2:** Table of echocardiographic features for ideal, challenging and relative contraindications for mitral edge-to-edge repair.

	**Ideal echo features**	**Challenging echo features**	**Relative echo contraindications**
Location of pathology	Segment 2	Segments 1 or 3	• Body of leaflet (i.e., perforation or cleft/deep fold)
Calcification	None	• Mild, outside grasping zone • Extensive annular calcification	• Severe calcification at site of grasping zone
Mitral valve area/gradient	• >4 cm^2^ • ≤4 mm Hg	• >3.5 cm^2^ and <4 cm^2^ with small BSA or mobile leaflets • ≥4 mm Hg	• <3.5 cm^2^ and ≥4 mm Hg
Grasping zone length	• >10 mm	• 7–10 mm	• <7 mm
Functional MR	• Normal thickness and mobility • Coaptation depth <11 mm	• Carpentier IIIB (restricted) • Coaptation depth >11 mm	• Carpentier IIIA (rheumatic thickening and restriction)
Degenerative MR	• Flail width <15 mm • Flail gap <10 mm	• Flail width <15 mm with large valve area and option for >1 MitraClip • Flail gap >10 mm with possibility of adjunctive measures	• Barlow's disease with significant regurgitation segments 1–3
Other pathology		• Annuloplasty ring with adequate mitral valve area and length • HOCM with systolic anterior motion • Extreme disease (markedly dilated annulus or EROA ≥70.8 mm^2^)	

*Reproduced with permission from Hahn RT. Transcathether Valve Replacement and Valve Repair: Review of Procedures and Intra-procedural Echocardiographic Imaging. Circ Res. 2016;119:341-56. BSA, indicates body area; EROA, effective regurgitant orifice area; HOCM, hypertrophic obstructive cardiomyopathy; and MR, mitral regurgitation*.

**Figure 2:**
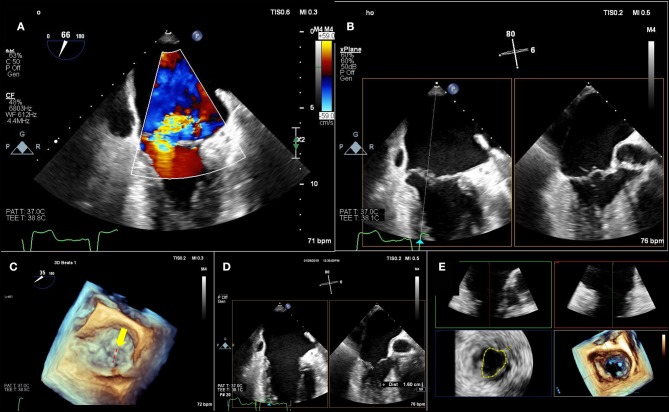
Pre-Mitraclip mitral valve anatomy characterization. **(A)** A large, medial regurgitant jet is demonstrated. **(B)** On bi-plane imaging of the region, medial anterior flail segment is demonstrated. **(C)** A 3-dimensional echocardiographic surgical view demonstrates A2-A3 scallop flail (yellow arrow) with a ruptured chord (red-dashed line). **(D)** Leaflet length in planned “grasping view” is measured and appears adequate for insertion into edge-to-edge repair device. **(E)** 3-Dimensional echocardiographic reconstruction demonstrates adequate baseline mitral valve area of 4.4 cm^2^, greater than the 4.0 cm^2^ cutoff.

For patient anatomic TEE screening, mitral anatomy should be completely described, including etiology of mitral regurgitation, specific scallop location(s) of mitral regurgitant jet(s), and leaflet qualities at these locations (thickening, calcification) (Figure 2). A biplane evaluation from the commissural view, interrogating the mitral valve across the commissures, is useful to localize anatomy at the grasping location and plan the “grasping view.” A wide coaptation gap may necessitate the XTR system, which has longer gripper arms for wider reach. MVA should be planimetered using 3D echocardiography multiplanar reconstruction during maximal or near maximal mitral valve opening in mid-diastole, taking care to measure at the leaflet tips. Care should be taken to reduce 3D volume size to maximize frame rates. Multi-beat or spliced imaging is not typically recommended, as this may create artifacts which will render MVA measurement inaccurate. Currently, 2 versions of the MitraClip are available, the XTR, and NTR. Table 3 lists specifications and considerations for each version. The XTR has a wider reach and longer clip arms than NTR. Although experience is limited thus far, the XTR is likely better for wide coaptation gaps with a higher risk of chordal entanglement for lesions at the commissures. Recent experience has been described using XTR in Barlow's disease (22) and as an adjunct after Cardioband (23) and transapical neochord implants (24). Early, compassionate use data have been published using a similar device (25), the Edwards Pascal, and the ongoing CLASP IID pivotal trial will provide further study.

**Table 3:** Mitraclip XTR vs. NTR specifications and preferred device for specific anatomical considerations.

**Specification**	**XTR**	**NTR**
Closed Clip Length (mm)	18	15
Grasping Width at 120° (mm)	22	17
Clip Arm Length (mm)	12	9
Desired leaflet insertion length (mm)	9	6
**Device preferred for anatomical consideration**		
Longer leaflet	X	
Large gap	X	
Redundant leaflet	X	
Leaflet calcification		X
Smaller mitral valve area		X
Mitral valve commissures		X

### Intra-Operative Imaging Guidance

**Figure 3:**
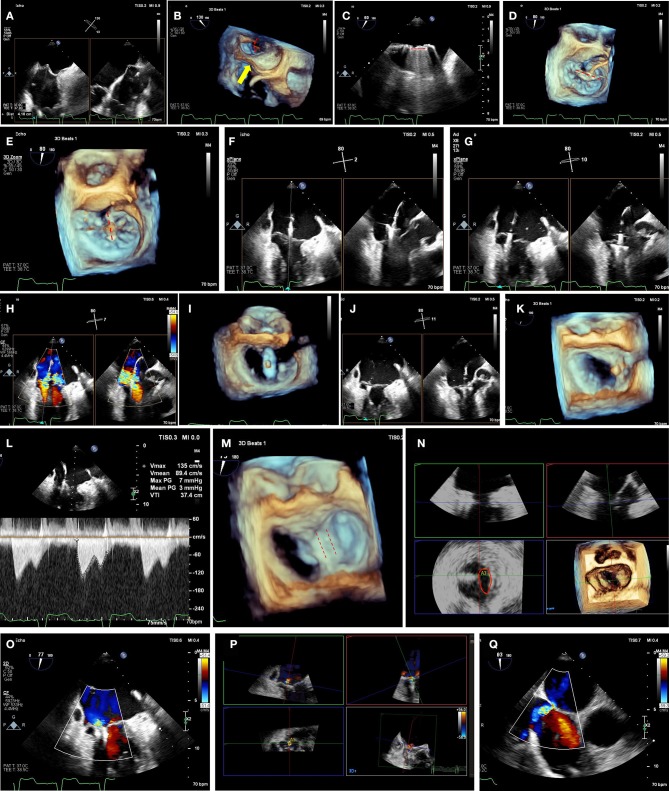
Mitraclip intra-procedural guidance. **(A)** Transseptal puncture should be performed in a mid-posterior location within the inter-atrial septum, 4.0-4.5 cm basal to the mitral annulus plane. Bi-plane imaging allows simultaneous visualization of the bicaval (typically 90–110°) and the 4-chamber (typically 0 or 180°, or orthogonal view from biplane imaging of bicaval view) view for localization of the puncture. **(B)** 3-dimensional echocardiographic imaging of needle tenting at the interatrial septum (yellow arrow) confirms trajectory across the mitral commissures (red dashed arch). **(C)** Extrusion of the mitraclip from the delivery sheath should be visualized to avoid injury from contact with the atrial wall. **(D)** After straddling the clip delivery system across the mitral commissures, the clip is opened while visualizing the 3D-TEE surgeon's view (left atrial perspective, aortic valve at 12 o'clock position) to check orientation (red-dashed line). In the image shown the clip needs rotation of 90° to achieve appropriate orientation perpendicular to commissures. **(E)** After clockwise rotation, the clip is oriented in planned clipping direction (red-dashed line) on the surgeon's view for A2-A3 flail segment. **(F)** Mitraclip position is confirmed from a bi-plane of commissural and 3-chamber views. On the 3-chamber views, clip arms are well-visualized. The biplane view is particularly useful in a non-central jet, as a traditional 3-chamber view may not demonstrate the grasping direction. Conversely, placing a biplane in the commissural view at the desired grasping location will provide the orthogonal grasping view. **(G)** As the mitraclip passes into the left ventricle, stable orientation and location should be confirmed. **(H)** Clip location is confirmed at the mitral regurgitation jet location by bi-plane color Doppler imaging. **(I)** By reducing gain settings, the clip orientation in the left ventricle can be confirmed by 3-dimensional echocardiographic imaging. **(J)** As the clip is pulled back toward the mitral leaflets, the insertion of each leaflet into each clip arm should be visualized. As the clip arms are fully closed, live color Doppler imaging may also be used to confirm reduction of the mitral regurgitation jet. **(K)** A tissue bridge is seen on 3-dimensional echocardiographic imaging after leaflet clipping, confirming adequate grasp. **(L)** Transmitral continuous-wave Doppler should be used to assess increase in gradients. If there is an unacceptable increase, clip can be released and repositioned or removed if still attached to delivery system. **(M)** After second clip is placed, both clips can be seen (red-dashed lines) on 3-dimensional echocardiographic imaging, with tissue bridge indicating bileaflet grasp of each clip. **(N)** Multiplanar 3-dimensional reconstruction allows for planimetry of each mitral orifice (red oval). Orifice areas are added together to calculated total mitral valve area after clip placement. If area is inadequately small and clip is still attached to delivery system, clip may be withdrawn and/or repositioned. **(O)** Post-clip mitral regurgitation is qualitatively mild by 2-dimensional color Doppler imaging. **(P)** 3-dimensional color Doppler multiplanar reconstruction allows alignment of mitral regurgitant jet(s) and direct planimetry of vena-contracta area (lower left panel), adding multiple jet areas together if needed. **(Q)** After withdrawal of delivery system and guide catheter, an iatrogenic atrial septal defect is visualized with left-to-right shunt by color Doppler. In the absence of right-to-left shunting with drop in oxygen saturation, post-procedural atrial septal defects generally do not require closure.

Intra-operative imaging guidance for MitraClip is predicated upon 2D and 3D TEE imaging. The procedural plan based on screening and intra-operative diagnostic TEE, including location and numbers of clips proposed, should be discussed between the interventional imager and interventional cardiologist. A qualitative and quantitative re-evaluation of the mitral regurgitation intra-procedurally, prior to MitraClip placement, is needed to establish a baseline for comparison at the end of the case. A step-by-step overview of imaging-based procedural steps is outlined in Figure 3. The initial, and possibly most important, step, is imaging guidance of the transseptal puncture. The ideal location for transseptal puncture is mid-fossa in a bicaval view, and ~4–4.5 cm basal from the mitral annulus as visualized in the 4-chamber view. The anterior-posterior rotation of the catheter typically determines the height above the annulus with more posterior positions gaining height. The exact height above the annulus for the transseptal puncture is determined by the planned positioning of the device: less height is required for a lateral defect, and more height is required for a medial defect as deflecting the system toward the mitral annular plane from lateral to medial will move the Mitraclip beyond the mitral annulus if the puncture is too close to the mitral annulus. The superior-inferior position of the catheter determines the position relative to the commissure. Aligning the puncture with the medial commissure facilitates positioning of the device anywhere along the mitral coaptation line. With atrial dilation that is commonplace in this patient population, the location of the interatrial septum to the mitral commissures may be distorted. This distortion is difficult to appreciate on 2D TEE imaging alone; thus, 3D TEE confirmation of transseptal puncture location is recommended (Figure 3). Caution is advised as one approaches the borders of the heart, so as not to puncture outside of the cardiac chambers (26). Altiok et al. showed the utility of 3D TEE for Mitraclip procedural guidance by having an interventional cardiologist evaluate 2D vs. 3D TEE for the procedural steps (27). 3D TEE was graded as superior for to 2D TEE for 9 of the 11 procedural steps studied, including transseptal puncture, guidance of the delivery system toward the mitral valve, positioning of the delivery system above the mitral valve, adjustment of the orientation of opened clip arms in relationship to the commissures, visualization of inserted clip position relative to the residual regurgitant jet after clip arm closure, and safe removal of the clip delivery system from the left atrium. 3D TEE was graded as inferior to 2D TEE only for leaflet grasping and evaluation of leaflet insertion. However, more contemporary clinical experience has shown the utility of visualizing a tissue bridge across the grasped leaflets by 3D echocardiography (Figure 3).

### Post-implant Assessment

Recent guidelines delineate the many pitfalls of routine measures of MR severity following percutaneous edge-to-edge repair (28). Importantly, PISA is not recommended given the assumption of a hemispheric flow convergence, the frequent presence of multiple MR jets and possible acoustic shadowing by the device. Quantitative Doppler also is limited by the presence of the edge-to-edge device and the presence of a double orifice. Thus, despite the multiple limitations of color Doppler parameters, flow convergence, vena contracta width, and jet area must be part of the multi-parametric assessment which should also include:

Mitral and pulmonary vein inflow patterns, change in forward stroke volume, and continuous wave jet profile. The primary quantitative parameter still valid following an edge-to-edge repair is three-dimensional color Doppler direct planimetry of the vena contracta areas (19, 29, 30). 3D color Doppler multiplanar reconstruction may be the method of choice to evaluate residual vena contracta area. In a recent study, final intra-procedural 3D color Doppler planimetered vena contracta area <27 mm2 was associated with improved New York Heart Association functional class at 30 day follow-up (30). In another study by Altiok et al. left atrial and left ventricular volumes were significantly more reduced at 6 month follow-up in patients in whom 3D-TEE measured vena contracta area was reduced by >50% (19).

Similarly to the pre-procedural evaluation, residual mitral valve orifice area should be planimetered on 3D multiplanar reconstruction, with each orifice area measured and added to evaluate the total orifice area. If significant mitral stenosis is present by planimetered mitral valve orifice area or transmitral gradient while the clip is attached to the delivery system, the clip can then be released, repositioned and/or withdrawn.

After clips are released from the delivery system, tissue grasp should be reconfirmed, and residual regurgitation, mitral valve orifice area, and transmitral gradient by continuous-wave Doppler should be evaluated. Complications should be monitored on TEE imaging during and after the procedure, including pericardial effusion, clip detachment, and damage to the leaflets or subvalvular apparatus.

## Cardioband

The Cardioband mitral annuloplasty system has been described in detail elsewhere (31). In short, this system is composed of a series of metal anchors contained within a Dacron band which are implanted in step-wise fashion around the circumference of the mitral annulus. After the last anchor is placed, the device is cinched for annular reduction. This mimics surgical annuloplasty. Early experience has been promising (31, 32). Recently, the single-arm, multi-center European trial showed reasonable safety and efficacy with a significant reduction in septolateral diameter by echocardiography from 3.7 ± 0.4 to 2.6 ± 0.4 (*p* < 0.01) immediately post-procedure, which was maintained at 1 year follow-up (32). The Annular Reduction for Transcatheter Treatment of Insufficient Mitral Valve Pivotal randomized, controlled clinical trial comparing a combination of Cardioband repair and guideline directed medical therapy against guideline directed medical therapy alone is ongoing in the United States.

### Procedural Planning

**Figure 4:**
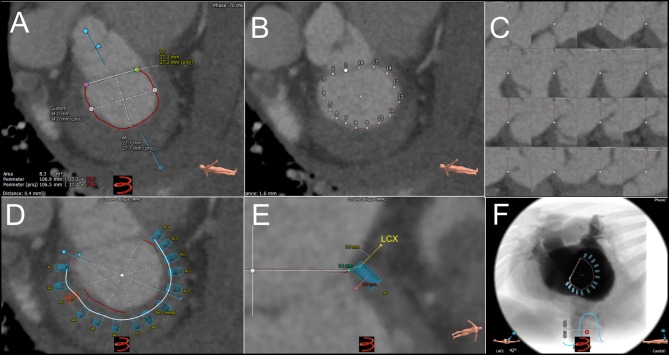
Pre-procedural cardiac computed tomography virtual anchor planning for cardioband. **(A)** Cubic-spline interpolation of multiple points is used in a semi-automated workflow to calculated projected mitral annulus perimeter excluding the trigone-to-trigone distance, which is used for sizing of Cardioband. **(B)** Control points of cubic-spline interpolation are visualized and can be adjusted on the short-axis, or long axis views **(C)**. **(D)** Anchor planning can be performed from trigone to trigone to visualize the trajectory of band. In this image, 14 anchors are planned with a potentially unsuitable location marked as a red anchor. **(E)** Each virtual anchor can be visualized (blue rectangle), and projected distances from anchor head to mitral annulus (turquoise line), mid-anchor to left ventricle (pink line), and nearby blood vessels such as the left circumflex coronary artery (yellow line) can be measured. **(F)** Projected fluoroscopic views can be planned.

All patients receiving mitral Cardioband should undergo preprocedural TEE (with 2D and 3D imaging) as well as MDCT. Mitral regurgitation etiology and severity should be assessed on TEE imaging as previously described. Preprocedural anatomic planning is primarily based on MDCT. MDCT analysis for Cardioband requires a dedicated software module such as those found on 3mensio (Pie medical imaging, Maastricht, Netherlands). Posterior mitral annulus perimeter (excluding trigone-to-trigone distance) should be measured using a cubic-spline interpolation measurement from a semi-automated workflow (Figure 4). The length of the implant and number of anchors is determined from the perimeter measurement (Table 4). Width of the annular shelf should be evaluated around the proposed implantation circumference on MDCT and TEE to determine whether adequate tissue for anchoring is present. When modeling device implantation on MDCT, anchor implantation angle of 30–60° is expected. Acceptable parameters are thought to include mid-anchor to LV distance of >4 mm and anchor head to mitral leaflet hinge point distance of <8 mm. These imply enough tissue in the mitral annulus for implantation as well as adequate distance from the leaflet to ensure annular reduction. Given the proximity of the left circumflex coronary artery to the mitral annulus, an anchor to left circumflex distance of >2.5 mm is desired. The coronary sinus location should also be noted, as it may also be injured if close to the proposed anchoring location. The optimal position for transseptal puncture may be planned for each patient from CT analysis.

**Table 4:** Edwards cardioband sizing chart.

**Deployment length by CT (mm)**	**Implant size**	**Total anchors required**
73–80	A	12
81–88	B	13
89–96	C	14
97–104	D	15
105–112	E	16
113–120	F	17

*CT, computed tomography*.

### Intra-Procedural Guidance

**Figure 5:**
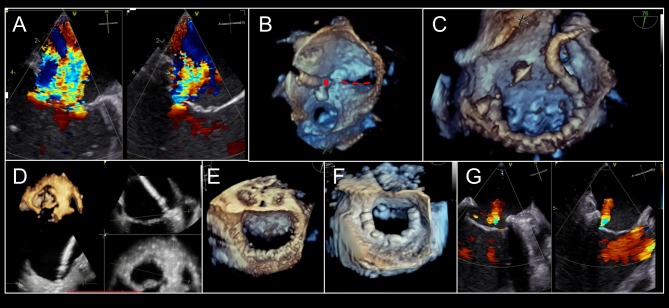
Intra-procedural echocardiographic guidance for cardioband procedure. **(A)** Severe, functional mitral regurgitation is shown in biplane, color Doppler imaging. **(B)** After transseptal puncture, the first anchor (red circle) is implanted adjacent to the lateral commissure (red-dashed line). **(C)** Cardioband implantation continues adjacent to P3 scallop. **(D)** At each anchor implantation, multiplane 3D imaging is helpful to locate position of anchor insertion within the mitral annulus, with adequate distance from leaflet. **(E)**. Completed Cardioband pre-cinching is shown. **(F)** After cinching, annuloplasty reduction is achieved. **(G)** Post-implant mitral regurgitation appears mild by qualitative assessment. 3-dimensional color Doppler multiplanar reconstruction may be used to planimeter vena contracta area. *Images courtesy of Dr. Florian Deuschl*.

Intra-procedural Guidance of mitral Cardioband is based on 2D and 3D-TEE, and fluoroscopy. Intracardiac echocardiographic imaging may play an increasing role as technology improves. As with Mitraclip, an initial intra-procedural baseline assessment of mitral regurgitation severity, mitral valve orifice area, and transmitral gradients should be performed for direct comparison post-implant. Additionally, baseline mitral annulus dimensions should be recorded for direct post-implant comparison. Transseptal puncture should be 3.0–4.5 cm above the mitral annular plane, and entering the atrium across the anteroseptal commissure on 3-dimensional TEE surgical (en-face) view. The first anchor is implanted as anterior as possible to the lateral commissure near the trigone. The positioning of the guide toward the first anchor location can be performed using 3D-TEE (Figure 5). Once the correct location is reached, 2D-TEE single and live multiplane imaging should then be used to confirm delivery system location upon the mitral annulus, with care taken to avoid implantation in the base of the leaflet. Each anchor is progressively deployed from the first location posteriorly until the medial commissure/trigone is reached. Before the release of each anchor, a pull test is performed. TEE and fluoroscopic confirmation of tissue anchoring is visualized. Once all anchors are deployed and confirmed and the implant contracted, a post-procedural TEE assessment of mitral annulus dimensions and mitral regurgitation severity should be performed. 3D-TEE multiplanar reconstruction of mitral annulus dimensions, mitral valve orifice area and regurgitant orifice area is ideal for direct comparison to pre-procedural measurements. As progressively tighter levels of cinching may be performed, these parameters should be assessed after each cinch to ensure adequate reduction of MR without excessive reduction in mitral valve area and/or excessive increase in transmitral gradients.

## Conclusions

The newly established percutaneous mitral valve repair technologies rely heavily on multimodality cardiac imaging for pre-procedural patient selection, as well as for intra-operative imaging guidance. Cardiac imaging will continue to play a critical role in the success of these procedures.

## Disclosure

OK reports speaker fees from Edwards Lifesciences; consulting for Jenavalve and Cephea Valves. RH reports speaker fees from Boston Scientific Corporation and Baylis Medical; consulting for Abbott Structural, Edwards Lifesciences, Gore & Associates, Medtronic, Navigate, Philips Healthcare and Siemens Healthcare; non-financial support from 3mensio and GE Healthcare; is Chief Scientific Officer for the Echocardiography Core Laboratory at the Cardiovascular Research Foundation for multiple industry-sponsored trials, for which she receives no direct industry compensation.

## Author Contributions

OK and RH both conceived the design of the document and approved the final document. OK drafted the document with significant edits and additions made by RH.

### Conflict of Interest Statement

The authors declare that the research was conducted in the absence of any commercial or financial relationships that could be construed as a potential conflict of interest.

## Abbreviations

MDCT: multi-detector row computed tomography
MR: mitral regurgitation
MVA: mitral valve area
TEE: transesophageal echocardiography
2D: 2-dimensional
3D: 3-dimensional.
